# Thioridazine Enhances Cisplatin-Induced DNA Damage in Cisplatin-Resistant Human Lung Cancer Cells

**DOI:** 10.1155/2022/3702665

**Published:** 2022-03-30

**Authors:** Yuanyuan Luo, Tinghe Yu, Xinya Li, Guanhua Qian

**Affiliations:** Laboratory of Obstetrics and Gynecology, The Second Affiliated Hospital, Chongqing Medical University, Chongqing 400010, China

## Abstract

Thioridazine was used to sensitize cisplatin against cisplatin-resistant human lung cancer cells. Cells received thioridazine, cisplatin, or both drugs (the combination). Thioridazine synergized cisplatin to increase percentages of dead and apoptotic cells. DNA damage was detected using the comet assays; the combination led to the highest alkaline- and neutral-comet percentages, demonstrating exacerbation of both single- and double-strand breaks. After thioridazine treatment, levels of glutathione, and BRCA2, RAD51, and ERCC1 proteins were decreased. These data manifested that thioridazine decreased the capacities of detoxification and DNA repair, thereby enhancing cisplatin-induced DNA damage in resistant cells.

## 1. Introduction

Lung cancer remains one of the most lethal malignancies worldwide, and cisplatin (DDP) is the first-line agent; however, the development of chemoresistance decreases the therapeutic responses and ultimately results in treatment failure [1–3]. Intracellular DDP is hydrated to generate the active forms, which induce DNA damage to cause apoptosis [3]. DDP resistance is multifactor, including drug influx and efflux, detoxification, DNA repair, and apoptosis malfunction [4–7]. Consequently, overcoming resistance is yet a challenge.

Thioridazine (THD) is commonly an antipsychotic drug. Recent data have demonstrated that THD can inhibit the proliferation of certain cancer cells (such as ovary, lung, uterine cervix, esophagus, melanoma, glioblastoma, and liver) [8–11]. THD binds to dopamine receptors on the cell membranes to activate biological reactions, i.e., THD can modulate the behavior of cells expressing dopamine receptors [12]. Our previous studies have indicated that THD can sensitize DDP against chemoresistant human lung cancer cells via enhancing apoptosis [10]. For cytotoxicity of DDP, DNA damage is the upstream event of apoptosis. However, how THD modulates DNA damage attributable to DDP has not been elucidated yet.

The aim of this study was to explore mechanisms of chemosensitization in resistant lung cancer cells from the perspective of DNA damage. Preliminary data indicated that THD enhanced DDP-induced DNA insults via modulating detoxification and DNA repair.

## 2. Materials and Methods

### 2.1. Cells

Human lung cancer cell line A549 and the chemoresistant subline A549/DDP were cultured in RPMI-1640 medium (GIBCO) supplemented with 10% fetal bovine serum (Biol. Ind., Kibbutz Beit-Haemek, Israel), at 37°C and 5% CO_2_. DDP (2.0 mg/mL; Qilu Pharm. Co. Ltd., Jinan, China) was added to the medium of A549/DDP to maintain the resistance phenotype. To avoid interferences of residual DDP, A549/DDP cells were transferred to DDP-free medium for 5 days before experiments [13, 14].

### 2.2. Cell Viability

Cells (5000 cells/well) were seeded on a 96-well plate and then were treated with THD (0, 5, 10, 15, 20, 25, 30, 35, and 40 *μ*M; Sigma-Aldrich, Darmstadt, Germany) or DDP (0, 5, 10, 20, 40, 80, 160, and 320 *μ*M) for 24 h. Drugs were washed away, and then, cells were cultured in complete medium. Cell vitality was detected at 24 h (i.e., 48 h after administering drugs) using a CCK-8 assay (Dojindo Lab., Kumamoto, Japan), and the half-maximal inhibitory concentration (IC_50_) was calculated.

Based on the cell survival percentages, 20 *μ*M THD and 40 *μ*M DDP were adopted in the combination regimen. Cells were subjected to THD and/or DDP for 24 h, and the viability was determined at 0, 24, and 48 h (i.e., 24, 48, and 72 h after administering drugs).

### 2.3. Detection of Apoptotic Cells

Apoptosis were determined using the Annexin V assay (Keygen Biotech., Nanjing, China) at 24 h after administering drugs. Apoptotic cells were the sum of early (V^+^/PI^−^) and late (V^+^/PI^+^) apoptotic cells.

### 2.4. Assessment of the Combination Effect

The combination index (CI) was calculated using percentages of dead cells (1-survival percentage), thereby determining the interaction between THD and DDP.

(1) $$\begin{array}{c} \text{CI}=\frac{E_{A+B}}{\left(E_{A}+E_{B}-E_{A}\times E_{B}\right)}. \end{array}$$

*E*_*A* + *B*_ was the effect of combination, and *E*_*A*_ or *E*_*B*_ was the effect of a single drug. A CI value of >1.15 indicated synergism, and 0.85–1.15 was addition [15].

### 2.5. Concentration of Glutathione (GSH)

The intracellular levels of GSH and GSSG were determined at 24 h after administering drugs using a kit (Beyotime Biotechnol., Shanghai, China).

### 2.6. Measurement of Reactive Oxygen Species (ROS)

Cells were treated as above and then incubated with 10 *μ*M dichlorofluorescein diacetate (DCFH–DA) (Beyotime Biotechnol., Shanghai, China) in darkness at 37°C for 30 min. ROS was determined with fluorescent spectrophotometry. *λ*_ex_ was 488 nm, and *λ*_em_ was 525 nm [16].

### 2.7. DNA Damage Detected with Comet Assays

Comet assays were performed to detect DNA damage at 24 h after administering drugs. The alkaline assay detected both single- (SSB) and double-strand break (DSB), and the neutral assay detected DSB. The percentage of comet-formed cells reflected the level of DNA damage [17].

### 2.8. Western Blot

Cells were harvested at 24 h after administering drugs. Proteins were extracted using the RIPA kit (Beyotime Biotechnol., Shanghai, China). Proteins (40 *μ*g/well) were separated by sodium dodecyl sulfate-polyacrylamide gel electrophoresis and transferred onto a polyvinylidene fluoride membrane. Rabbit antibodies (Abcam, Cambridge, UK) against BRCA2 (polyclonal), RAD51 (monoclonal), ERCC1 (monoclonal), and *β*-actin (monoclonal) were used. Rat-anti-rabbit IgG antibody (Abcam, Cambridge, UK) was the secondary antibody. Proteins were visualized by an enhanced chemiluminescence kit (Pierce Biotechnol, Rockford, USA). *β*-actin was the reference to quantify the expression level of a target protein.

### 2.9. Statistical Analysis

The IBM SPSS 26.0 (IBM, USA) software was used for statistical analyses. Analysis of variance (ANOVA) was adopted and the least significant difference (LSD) was used for multiple comparisons. The critical value was set *p* < 0.05.

## 3. Results

### 3.1. THD Synergized DDP against Resistant Cells

**Figure 1:**
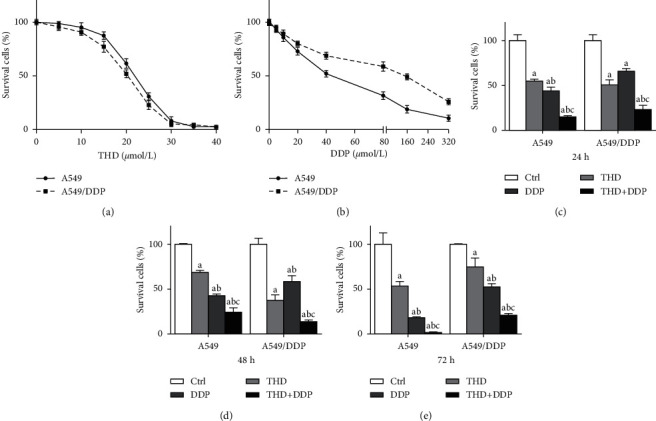
Cytotoxicity of THD and DDP in A549 and A549/DDP cells. Survival curves after exposure to THD or DDP with serials of concentration (a, b). Survival percentages at 24–72 h after administering to 20 *μ*M THD and/or 40 *μ*M DDP (c–e): the combination led to the lowest survival fractions. Data were mean ± standard deviation for 3 independent experiments. a vs. group ctrl, *p* < 0.05; b vs. group THD, *p* < 0.05; c vs. group DDP, *p* < 0.05.

Either DDP or THD dose-dependently deactivated A549 or A549/DDP cells (*p* < 0.001) (Figures 1(a) and 1(b)). Both cell lines displayed similar responses to THD, with IC_50_ of 20.91 and 18.54 *μ*M, respectively. For DDP, cell survival percentages in A549/DDP cells were higher than those in A549 cells, with IC_50_ of 45.44 and 116.92 *μ*M, respectively, confirming the resistance property of A549/DDP. Therefore, 20 *μ*M THD and 40 *μ*M DDP were chosen for the combination therapy.

The combination regimen (THD + DDP) led to the lowest cell survival percentage (A549: *p* < 0.001; A549/DDP: *p* < 0.001) (Figures 1(c)–1(e)). CI were 1.09 (1.07–1.12) in A549 cells (i.e., addition) and 1.18 (1.10–1.30) in A549/DDP cells (i.e., synergism). These data indicated that THD synergized DDP against resistant cells.

### 3.2. THD Enhanced Apoptosis Due to DDP

**Figure 2:**
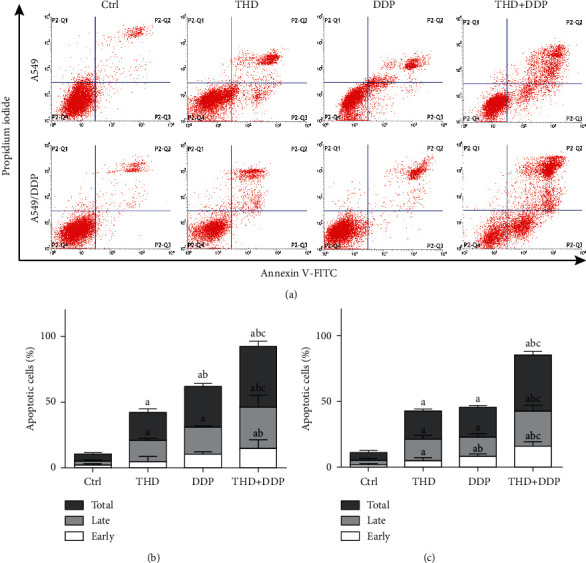
Apoptosis after treatments with THD and/or DDP in A549 (a, b) and A549/DDP (a, c) cells. The combination regimen caused the highest apoptosis percentage. Data were mean ± standard deviation for 3 independent experiments. a vs. group ctrl, *p* < 0.05; b vs. group THD, *p* < 0.05; c vs. group DDP, *p* < 0.05.

DDP led to a lower apoptosis percentage in A549/DDP cells compared with A549 cells (23.0 ± 1.3% vs. 31.1 ± 2.1%, *p*=0.040). The apoptosis percentage in the combination regimen was higher than that after receiving THD or DDP (A549: *p* < 0.001; A549/DDP: *p* < 0.001) (Figure 2). Late apoptosis had a higher weight. These data manifested that THD can enhance DDP-induced apoptosis.

### 3.3. THD Decreased GSH

**Figure 3:**
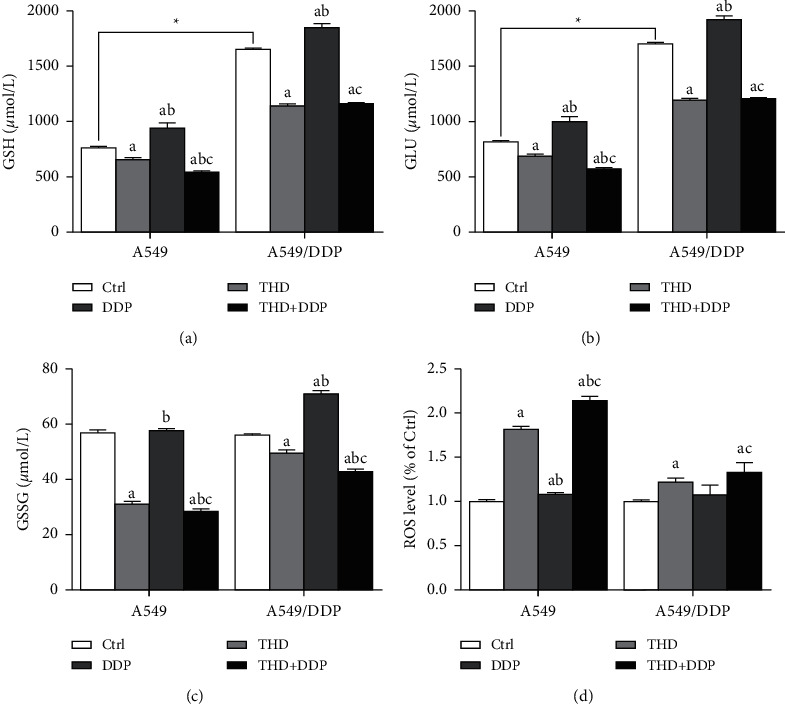
Levels of GSH and ROS. Total and reduced GSH were decreased after receiving THD or the combination regimen (a, b). ROS level (d): a higher level was detected after treatments with THD or the combination regimen. Data were mean ± standard deviation for 3 independent experiments. a vs. group ctrl, *p* < 0.05; b vs. group THD, *p* < 0.05; c vs. group DDP, *p* < 0.05. ^*∗*^*p* < 0.05.

The levels of total and reduced GSH in A549/DDP cells were higher than those in A549 cells (*p* < 0.001, *p* < 0.001). THD or the combination regimen decreased the levels of total and reduced GSH (A549: THD *p*=0.007, combination *p* < 0.001; A549/DDP: THD *p* < 0.001, combination *p* < 0.001), but increases were noted after DDP treatment (A549: *p* < 0.001; A549/DDP: *p* < 0.001) (Figure 3(a)). A higher ROS level was detected after THD treatment, with the highest level in cells receiving the combination regimen (A549: *p* < 0.001; A549/DDP: *p*=0.001) (Figure 3(d)). These data indicated that THD can decrease the intracellular level of GSH.

### 3.4. THD Enhanced DDP-Induced DNA Damage

**Figure 4:**
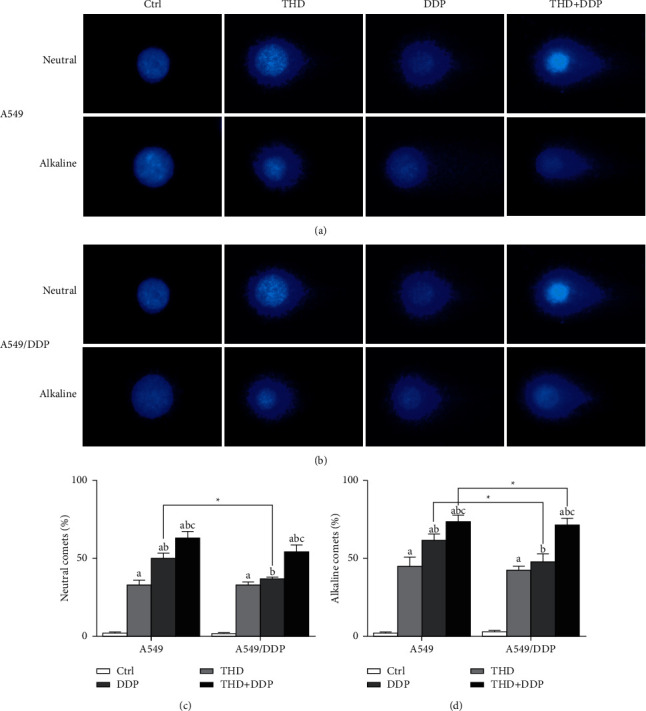
DNA damage after exposure to THD and/or DDP. Representative comet images (a, b). Comet percentages in the alkaline (c) or neutral (d) assay: the highest value was noted after receiving the combination regimen. Data were mean ± standard deviation for 3 independent experiments. a vs. group ctrl, *p* < 0.05; b vs. group THD, *p* < 0.05; c vs. group DDP, *p* < 0.05, ^*∗*^*p* < 0.05.

Alkaline and neutral assays demonstrated that THD, DDP, and the combination induced comet formation, and the combination regimen resulted in the highest comet percentage (A549: *p* < 0.001 for each; A549/DDP: *p* < 0.001 for each) (Figure 4). After DDP treatment, both the alkaline- or neutral-comet percentage in A549/DDP cells were lower than those in A549 cells (*p*=0.001; *p* < 0.001) (Figure 4).

**Figure 5:**
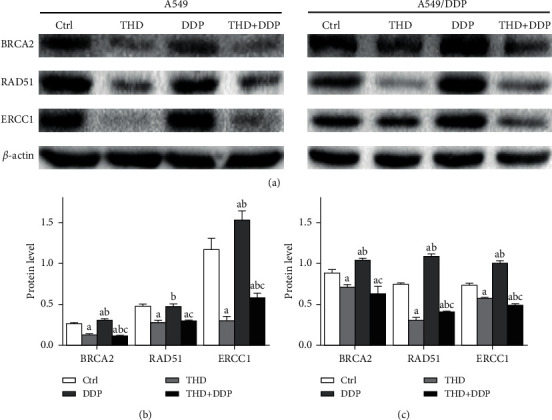
DNA-repair related proteins BRCA2, RAD51, and ERCC1 validated by western blot in A549 (a, b) and A549/DDP (a, c) cells. Levels of BRCA2, RAD51, and ERCC1 were decreased after receiving THD or the combination regimen but were increased after DDP treatment. Data were mean ± standard deviation for 3 independent experiments. a vs. group ctrl, *p* < 0.05; b vs. group THD, *p* < 0.05; c vs. group DDP, *p* < 0.05.

BRCA2 and RAD51 were key molecules for DSB repair, and ERCC1 was the critical protein in SSB repair [18]. Western blot indicated that the level of BRCA2, RAD51, and ERCC1 was decreased after treatment with THD or the combination of THD and DDP but was increased after DDP treatment (A549: BRCA2 *p*=0.001, RAD51 *p*=0.078, ERCC1 *p*=0.001; A549/DDP: BRCA2 *p*=0.007, RAD51 *p* < 0.001, ERCC1 *p* < 0.001) (Figure 5). These data demonstrated that THD enhanced DDP-induced DNA damage and can decrease the DNA repair capacity in resistant cells.

## 4. Discussion

DNA was the preferred target of DDP. DDP frequently attacked guanine and adenine to induce intra- and interstand crosslinks, causing SSB and DSB [19, 20]. Most SSB can be repairable and partial SSB would evolve into DSB; unrepairable DSB triggered apoptosis to deactivate cells [21]. Resistant cells had a higher capacity of DNA repair, thereby protecting cells from DNA damage [21]. These were consistent with the present data that the cell survival was higher and apoptosis and comet percentages were lower in A549/DDP cells than those in A549 cells. Therefore, chemosensitization due to THD was explored from the perspective of DNA damage in the present study.

Data on cytotoxicity and apoptosis accorded with the previous findings, verifying the sensitization effect of THD, i.e., data on DNA damage in this study were valuable [10]. Comet percentages in the combination group were higher than those in the DDP group, indicating that THD enhanced DNA damage induced by DDP. An exacerbation of DNA damage caused more cells undergo apoptosis. Here, the ratio of neutral- to alkaline-comet percentages was >76% in the combination therapy, i.e., most damage was DSB. In resistant ovarian cancer cells COC1/DDP, the ratio was <40% when using cyclosporin/ultrasound to sensitize DDP, or exposure to electric pulses [20]. These data indicated that the feature of DNA damage depended on cell type and the therapeutic means. Increasing the proportion of DSB favored apoptosis. Thus, THD modulated DNA insults, promoted apoptosis, and eventually synergized DDP.

DSB was repaired via homologous recombination (HR), where BRCA2 and RAD51 were essential molecules [22, 23]. ERCC1 was the key protein for nucleotide excision repair (NER) that repaired SSB [24, 25]. Lung cancer patients with a high expression level of those molecules in cancer tissues had poor therapeutic responses and shorter survival time [24]. The present data showed that THD decreased the level of these 3 proteins, indicating that both SSB and DSB repairs were suppressed. The persistence of SSB and DSB accumulated errors, resulting in cell death. Upregulation of these proteins was noted after DDP treatment, which was consistent with previous reports and was inductive expression [26]. DNA damage attributable to DDP activated the adaptive responses to protect cells. Consequently, the repair capacity can be actually decreased only when the downregulation effect surpassed the upregulation effect. In this study, the levels in the combination group did not exceed those in the THD group. This played a part in the highest comet percentages observed in the combination group.

Intracellular processes of DDP included activation (i.e., forming hydrated platinum) and inactivation. DDP was inactivated when binding with GSH and metallothioneins, and the conjugate was pumped out by GS-X [27]. A high intracellular level of GSH led to DDP resistance, and decreasing GSH improved the cells' sensitivity to DDP [28]. This verdict accorded with the present data that a higher GSH level was detected in A549/DDP cells. THD decreased the GSH level, thereby increasing the intracellular amount of active platinum. That THD reduced GSH via lowering the yield or accelerating the degradation should be elucidated. ROS was involved in the action of DDP, and a decrease in ROS related to resistance [29, 30]. ROS mediated cytotoxicity via lipid peroxidation. Here, the combination led to the highest ROS level, implying that THD can facilitate the ROS generation attributable to DDP. Additionally, GSH was an antioxidant that can scavenge ROS. THD decreased the GSH level, favoring intracellular accumulation of ROS.

Noticeably, for cell death and apoptosis, A549 and A549/DDP cells displayed similar responses to THD; THD also enhanced the action of DDP on A549 cells from the perspective of cell apoptosis and DNA damage. This can be an advantage, i.e., determining the DDP sensitivity prior to treatments was unnecessary. The present study was an in vitro trial, and therefore the aforementioned verdicts should be tested in vivo.

**Figure 6:**
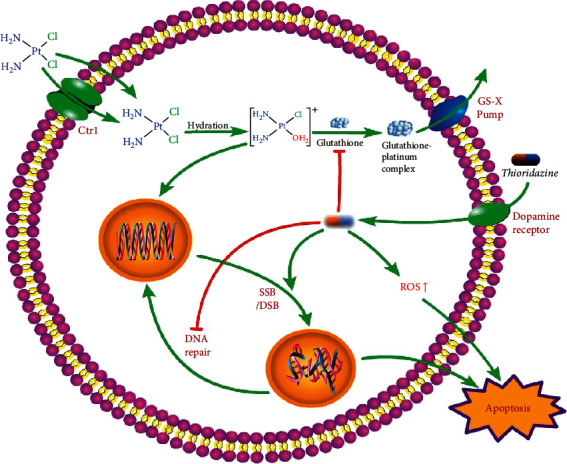
Illustration of mechanisms. THD enhanced DDP via decreasing detoxification and DNA repair.

In summary, THD reduced the GSH level to increase the intracellular amount of active platinum; THD downregulated the levels of ERCC1, BRCA2, and RAD51 proteins, thereby decreasing the DNA repair capacity. These effects enhanced DDP-induced DNA damage, promoted/exacerbated DSB, and eventually triggered apoptosis to deactivate resistant lung cancer cells (Figure 6).

## Data Availability

The data used to support the findings of this study are included within the article.
